# Investigating COVID-19 News Across Four Nations: A Topic Modeling and Sentiment Analysis Approach

**DOI:** 10.1109/ACCESS.2021.3062875

**Published:** 2021-03-01

**Authors:** Piyush Ghasiya, Koji Okamura

**Affiliations:** 1 Graduate School of Information Science and Electrical EngineeringKyushu University 12923 Fukuoka 819-0395 Japan; 2 Research Institute for Information Technology, Kyushu University 12923 Fukuoka 819-0395 Japan

**Keywords:** COVID-19, natural language processing, newspaper, machine learning, RoBERTa, sentiment analysis, topic modeling, Top2Vec

## Abstract

Newspapers are very important for a society as they inform citizens about the events around them and how they can impact their life. Their importance becomes more crucial and indispensable in the times of health crisis such as the current COVID-19 pandemic. Since the starting of this pandemic newspapers are providing rich information to the public about various issues such as the discovery of a new strain of coronavirus, lockdown and other restrictions, government policies, and information related to the vaccine development for the same. In this scenario, analysis of emergent and widely reported topics/themes/issues and associated sentiments from various countries can help us better understand the COVID-19 pandemic. In our research, the database of more than 100,000 COVID-19 news headlines and articles were analyzed using top2vec (for topic modeling) and RoBERTa (for sentiment classification and analysis). Our topic modeling results highlighted that education, economy, US, and sports are some of the most common and widely reported themes across UK, India, Japan, South Korea. Further, our sentiment classification model achieved 90% validation accuracy and the analysis showed that the worst affected country, i.e. the UK (in our dataset) also has the highest percentage of negative sentiment.

## I. Introduction

In December 2019, the outbreak of pneumonia with an unknown cause was discovered in Wuhan, Hubei Province, China. The virus that caused this outbreak was later found and named severe acute respiratory syndrome coronavirus 2 (SARS-CoV-2). In February 2020, the World Health Organization (WHO) called the disease caused by SARS-CoV-2 “COVID-19” [1]. According to Johns Hopkins Coronavirus Resource Center, this virus has infected more than 95.48 million people and taken the lives of more than 2.03 million people as of January 18, 2021 [2]. However, the end of this pandemic seems near as there are many vaccines candidates that are currently in phase III trials. F. Krammer in “SARS-CoV-2 vaccines in development” [3] and G. Forni and A. Mantovani in “COVID-19 vaccines: where we stand and challenges ahead” have discussed in detail the vaccines development process, different kind of vaccines, and the frontrunner vaccines candidates [4].

As the world is in the grip of the COVID-19 pandemic, Newspapers worldwide are extensively reporting about COVID-19 related news. This makes newspapers an incredible source to comprehend the social, economic, and political reality vis-a-vis this deadly pandemic in a particular society/country. Further, the practice of newsmaking is informed by a variety of cultural beliefs and ideological assumptions [5]. Thus, the product of newsmaking - news - is a social text, symbolically incorporating and recirculating those assumptions and beliefs and thereby reproducing social reality. Millions around the world lost their jobs due to the lockdown. India’s complete lockdown led to the migrant crisis of a monumental scale [6], many top economies of the world clocked negative GDP growth [7]. More than 330 companies filed for bankruptcies in the US alone last year and blamed COVID-19 in part for their demise [8].

Natural Language Processing (NLP) and its various techniques have gained prominence when it comes to process and analyze large amount of natural language data. NLP can be defined as: A field that combines linguistics and artificial intelligence (AI) to enable computers to understand human or natural language [9]. In present times, the criticality of NLP is further increased from the very fact that we are generating enormous amount of unstructured text data every day. Some of the most common and popular NLP techniques include named entity recognition [10], sentiment analysis [11], machine translation [12], topic modeling [13], and text summarization [14]. In the current COVID-19 crisis, one critical asset is to identify as much information about the problem as possible. Any information that is available, which can be located, amassed, and understood, will facilitate the right decisions to be taken. NLP enables researchers to access information on topics such as global and regional spread, socio-economic impact of the disease, vaccine development, patient demographics and co-morbidities, and the national and international politics from sources such as scientific literature, policy documents, social media and news [15].

### A. Research Objective

There are two main objectives of this research: 1) To examine key topics and themes of English-language COVID-19 news articles across four countries and explore the trends. Further, we would perform a comparative analysis of these topics to find out the common themes. 2) To classify and analyze the associated sentiments of the COVID-19 news headlines. This would help us understand: i) comparison of COVID-19 news sentiments across four nations, b) whether there is a correlation between negative sentiment and the level of affectedness of a country, and c) representative sentiments of common themes in different countries.

### B. Contribution

• We extracted the most representative topics or themes from newspapers of the four countries and discussed the dominant discourse concerning COVID-19. In this way, we find out the widely reported issues or themes concerning COVID-19 in news media.
• Our RoBERTa sentiment classification model not only achieved validation accuracy of 90% but also produced better results than other traditional classifiers.
• As per the knowledge of the authors, this is the first study to “combine topic modeling and sentiment analysis methods to analyze COVID-19 news.”
• Our study provides interesting cross cultural insights into the COVID-19 reporting in the news media platform.

This article is organized as follows: in Section II the related works are being discussed, while Section III has presented the methodological framework for both topic modeling and sentiment classification. In Section IV the experiments, results and the analysis are presented and discussed. Limitations of this research is summarized in Section V. Lastly, Section VI concludes this research article with potential future research directions.

## II. Related Work

### A. Topic Modeling

Researchers from around the world are trying to understand various aspects of the COVID-19 pandemic. Since the identification of the COVID-19 disease in December 2019, numerous studies from various fields have already been published. NLP-based research that analyzes COVID-19 related material such as scientific articles, social media posts, and news is one example of the growing body of research related to COVID-19. Bai *et al.* [16] presents a topic evolution analysis of COVID-19 news articles from Canada. Liu *et al.* [17] used a digital topic modeling approach to analyze news media during the early stage of the COVID-19 outbreak in China. De Santis *et al.* [18] presented an infoveillance system for detecting and tracking relevant topics from Italian tweets during the COVID-19 event. Noor *et al.* [19] analyzed the public reactions to the novel Coronavirus (COVID-19) outbreak on Twitter.

### B. Sentiment Analysis and Emotion Detection

Samuel *et al.* [20] talked about COVID-19 public sentiment insight and machine learning for tweets classification. Imran *et al.* [21] analyzed tweets from six countries for cross-cultural polarity and emotion detection using deep learning method. Huang *et al.* [22] presented sentiment convolutional neural networks to analyze the sentiment of sentences with both contextual and sentiment information of sentiment words. Boon-Itt and Skunkan [23] investigated Twitter posts using sentiment analysis and topic modeling approach to find out the public perception during the COVID-19 pandemic. Das and Dutta [24] characterized public emotions and sentiments in COVID-19 environment in India. Barkur *et al.* [25] presents sentiment analysis of nationwide lockdown due to COVID-19 outbreak in India.

### C. Topic Modeling and Sentiment Analysis

All the works cited from [16] to [25] either performed topic modeling or sentiment analysis on COVID-19 data. Compared to that, there are very few research that has combined both topic modeling and sentiment analysis to investigate COVID-19 data. Chandrasekaran *et al.* [26] discussed topics, trends, and sentiments of tweets about the COVID-19 pandemic using tools such as LDA and VADER. Xue *et al.* [27] analyzed tweets to understand public discourse and sentiment during COVID-19 pandemic. They used Latent Dirichlet Allocation (LDA) method for topic modeling. Lastly, Xie *et al.* [28] explored public response to COVID-19 on Chinese micro blogging site Weibo with LDA topic modeling and sentiment analysis. Our research can be categorized along with the researches which combine topic modeling and sentiment classification methods to analyze COVID-19 data. However, the nature of our COVID-19 (news articles) and methods (top2vec for topic modeling and RoBERTa for sentiment classification) makes this research a welcome addition to comprehend cross-cultural COVID-19 news. According to the authors’ best knowledge ours is the first study which attempted to analyze COVID-19 news articles by applying topic modeling and sentiment analysis methods.

## III. Methodology

**FIGURE 1.**
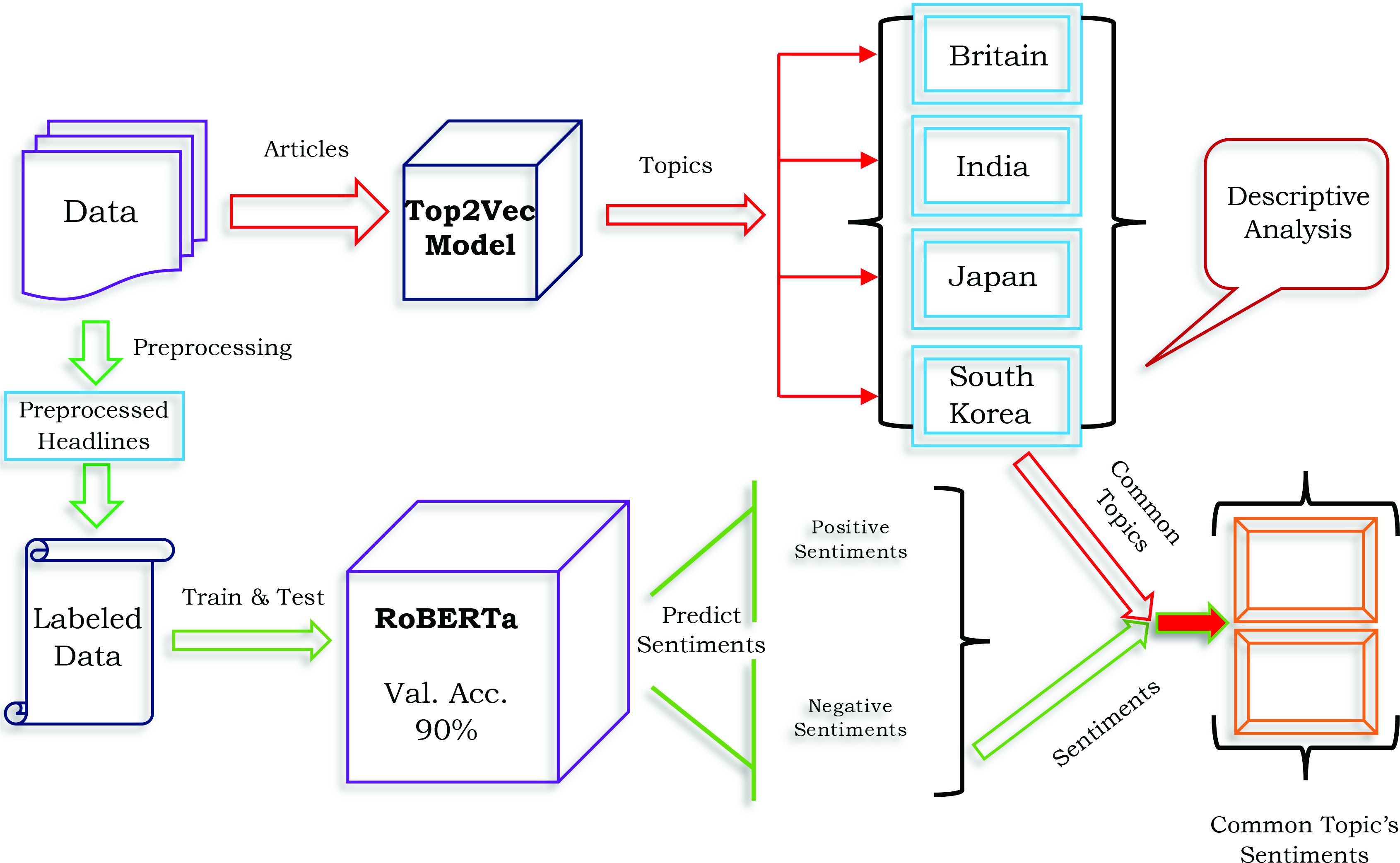
Workflow of Research Methodology.

As shown in Fig. 1, this research consist of two parts. In part one, we utilized the top2vec model to ascertain the most representative topics in each country’s dataset and analyze them in detail. Then sentiment analysis is the second part. This part can be further divided into two subsections. One section is about creating a labeled dataset using unsupervised machine learning methods. After that, next is to use RoBERTa to train and test our labeled dataset. After getting satisfactory validation accuracy, we predict the sentiments of collected headlines sentiments and the most common topics.

### A. Dataset

We searched and scraped articles with COVID-19 or Coronavirus keywords in the English language websites of eight major newspapers from four countries. The time-period for collection of both news headlines and articles is from January 1st, 2020 to December 1st, 2020. For web-scraping, we used the Beautiful Soup Python library. We removed the duplicate headlines and articles. In the top2vec model, preprocessing is not required. We trained, tested, and predicted the positive or negative sentiments on preprocessed headlines for sentiment classification [29]. Table 1 shows the newspapers and the collected COVID-19/Coronavirus related articles. All the Jupyter Notebooks developed for this research are available on paper’s GitHub repository.^1^^1^https://github.com/PiyushKyushu/COVID-19-News

**TABLE 1:** Dataset

Countries	Newspapers	No. of Articles
UK	The Daily Mail	23,821
India	Hindustan Times, The Indian Express	47,342
Japan	The Japan Times, Asahi Shimbun, Mainichi Shimbun	21,039
South Korea	Korea Herald, Korea Times	10,076
Total		102,278

### B. Topic Modeling and Top2Vec Model

Organizing, searching, and summarizing a large volume of text is a universal problem in NLP. Topic modeling is used when a large corpus of text cannot be judiciously read and sorted through by a person. A topic model can discover the latent semantic structure or topics present in the corpus. Top2Vec algorithm works on the assumption that many semantically similar documents are indicative of the underlying topic. It produces jointly embedded topic, document, and word vectors such that the distance between them represent semantic similarity. Moreover, unlike the traditional topic modeling methods such as LDA, it does not require preprocessing (removing stop-words, stemming, and lemmatization of text), and there is no need to have prior knowledge of existing topics to produce a good topic model [30]. In this way, top2vec is completely free from human decision making or interference except in choosing the parameters during training the model.

In the first step, jointly embedded document and word vectors are created using Doc2Vec [31]. This step would place documents close to other similar documents and the most distinguished words. The next step would use UMAP [32] to create a lower-dimensional embedding of document vectors. As document vectors in high dimensional space are very sparse, dimension reduction helps find dense areas. The third step would find dense areas of documents using HDBSCAN [33]. Finally, each dense area calculates the centroid of document vectors in the original dimension, i.e., the topic vector [34].

Though top2vec provides an option to use pretrained embedding models such as universal-sentence-encoder for generating joint word and document embeddings, but we use our own data to create topic models. We used ‘deep-learn’ parameter which generate best quality vectors but take significant time to train. For example it took around 16 hours on our system to train top2vec model on Indian dataset (largest dataset). Once the model is produced, it provides various information such as topic size, topic words, topic number and topic score etc. Topic size shows the number of documents most similar to each topic while for each topic the top 50 words (we have only presented top 10 words in Table 4, 5, 6, and 7) are returned, in order of semantic similarity to the topic. Topic score – for each topic is the cosine similarity to the search keywords. The higher the topic score, the most representative that topic would be for the searched keyword. This feature of top2vec is used in this research to find the most representative topic using keywords. The word cloud in Fig. 4, 5, 6, and 7 are also generated using the top2vec semantic search feature. Lastly, based on our understanding of the topic’s specific keywords (all 50 words) we labeled these topics.

**TABLE 2:** Sample Labeled Headlines With Sentiment

Headlines	Labeled Sentiment
Coronavirus crisis: 100,000 dead in 101 days, half of them in just a week	Negative
Coronavirus leading to heavy job losses among vulnerable workers in Japan	Negative
Covid-19 vaccine tracker, August 27: Moderna shot promises to be equally effective in older patients	Positive
New Zealand is now free of COVID-19	Positive

**TABLE 3:** No. of Topics Discovered From Top2Vec Model

Countries	No. of Articles	No. of Topics from Top2Vec Model
UK	23,821	308
India	47,342	402
Japan	21,039	255
South Korea	10,076	127

**TABLE 4:** UK’s Top 10 Topics With Topic Size, Topic Words, and Topic Label

No.	Topic Size	Top 10 Words	Topic label
1	369	Coma, Newborn, Neonatal, Ventilator, Mother, Ward, Sedate, Heartbreaking, Induce, Caesarean	Maternity during Coronavirus Pandemic
2	363	Pupils, Teachers, Education, Williamson, DFE, Exams, GCSES, NEU, Teach, ASCL	Education related News
3	305	Melburnians, Andrews, Victorians, Bourke, Melbourne, Victorian, Caregiving, Daniel, Victoria, Cautions	Australia related News
4	298	Sunbelt, Midwest, Tracking, IHME, Metrics, Surpass, Arizona, Tennessee, Texas, Nationally	US related News
5	275	ONS, England, Statisticians, Statistics, Data, Figure, Pendle, Oldham, Blackburn, Darwen	Office for National Statistics (ONS) related
6	268	EMS, Javits, Elmhurst, Epicenter, USNS, York, FEMA, Cuomo, Sinai, EMTS	US especially New York related News
7	248	Sunak, Chancellor, Rishi, Furlough, Borrow, Wag, Retention, Treasury, Economy, Fiscal	Chancellor (FM) and Eco policies during Pandemic
8	246	Ardern, Jacinda, Zealanders, Kiwis, Zealand, Auckland, Kiwi, Wellington, Bloomfield, Tasman	New Zealand related news
9	244	Job keeper, Jobseeker, Centrelink, Unemployed, Subsidies, Frydenberg, Fortnightly, Newstart, Dole, Treasurer	Unemployment and Jobs related
10	240	Coles, Pasta, Shelve, Flour, ALDI, Supermarket, Rice, Mince, Packets, Shelves	Panic Buying During Lockdown

**TABLE 5:** India’s Top 10 Topics With Topic Size, Topic Words, and Topic Label

No.	Topic Size	Top 10 Words	Topic Label
1	1278	Cricket, ODI's, ODI, Batsman, ESPNcricinfo, Cricketing, IPL, Cricketers, Pacer, Overs	Cricket and IPL
2	658	Trump, Democrat, Americans, Republican, Biden, Arizona, Delaware, Carolina, Florida, Donald	US related
3	623	Ludhiana, Jalandhar, Faridkot, Gurudaspur, Hoshiarpur, Amritsar, Sangrur, Bagga, Patiala, Moga	Punjab related news
4	610	Accused, Complainant, Murder, Allegedly, SHO, Arrested, IPC, FIR, Complaint, Incident	Law and Order during Pandemic
5	605	Vaccine, Astrazeneca, Vaccines, Doses, Oxford, Trails, AZD, Moderna, Pfizer, m-RNA	Vaccine Development
6	500	Classes, Classroom, Teachers, Learning, Worksheets, Teaching, Curriculum, Schools, Syllabus, School	School Education during Pandemic
7	431	RBI, Contraction, GDP, Liquidity, Monetary, Economy, Shaktikanta, Growth, Outlook, Fiscal	RBI and Economy related
8	423	Apr, Myself, Pdt, Chores, Enjoying, Adds, My, Me, Things, Mom	Celebrities on Social Media During Lockdown
9	388	Semester, UGC, Varsity, Universities, Examinations, Semesters, Academic, Varsities, Chancellors, Exams	University Education during Pandemic
10	380	ICMR, PCR, RT, Polymerase, Confirmatory, Rapid, Diagnostic, Antigen, Laboratories, Testing	Covid-19 testing

**TABLE 6:** Japan’s Top 10 Topics With Topic Size, Topic Words, and Topic Label

No.	Topic Size	Top 10 Words	Topic Labels
1	409	Investors, Stocks, Dow, NASDAQ, Gains, Benchmark, Composite, Markets, Wall, Treasury	Global Stock Exchanges News
2	382	Decliners, Section, Shares, Advancers, Yen, Brokers, Outnumbered, Nikkei, Fetched, Unchanged	Nikkei News
3	363	IOC, Bach, Olympics, Olympic, Organizing, Postponement, Athletes, Games, Federations, Paralympics	Tokyo 2020 Games Postponement
4	314	Eiju, Inpatients, Outpatients, Taito, Man, Hospital, Admitted, Woman, Hospitalized, Outpatient	COVID-19 Cases in different prefectures
5	293	KCDC, Seoul, Sye, Daegu, Kwon, Kyun, Eun, Gyeongsang, Kyeong, Itaewon	South Korea News
6	259	Biden, Joe, Nominee, Hillary, Kamala, Harris, Clinton, Sanders, Democrats, Delaware	US Election
7	258	Declaration, Prefectures, Pachinko, Governors, Emergency, Parlors, Hyogo, Requests, Nishimura, Outings	Declaration of Emergency in Japan and related news
8	251	Globalization, Democracies, Societies, Superpower, Technological, Authoritarian, Geopolitical, Multilateral, Nationalism, Alliances	Politics over Coronavirus Pandemic
9	241	Pyongyang, Jong, Kim, Un, North, KCNA, Korean, Kaesong, Koreas, Korea	North Korea News
10	240	Elementary, Education, School, Schools, Teachers, Classes, Hagiuda, Junior, Academic, Students	School Education amid Pandemic

**TABLE 7:** South Korea’s Top 10 Topics With Topic Size, Topic Words, and Topic Label

No.	Topic Size	Top 10 Words	Topic Labels
1	284	Supplementary, Budget, Extra, Fiscal, Budgets, Households, Handouts, Earners, Bill, Relief	Various Economic Relief during Pandemic
2	251	Syndicate, Europeans, Eurozone, Economies, Globalization, Geopolitical, Continent, Crises, Policymakers, Africa	Geopolitics During Pandemic
3	244	Stores, Shopping, Store, Mart, Shinsegae, Customers, Retailers, Mall, Items, Lotte	Pandemic's impact on Retail and Shopping
4	210	Monetary, BOK, Contraction, Forecast, Economy, Slashed, Outlook, Shrink, Projection, Growth	Lower GDP forecast due to Pandemic
5	205	Untraceable, Sporadic, Cluster, Infections, Surrounds, Cases, Traced, Area, Caseload, Digits	Coronavirus Cluster Cases
6	191	League, Matches, Season, Teams, Football, Players, Champions, Stadium, KBO, Match	Various Sports League in Korea amid Pandemic
7	178	Smartphone, Smartphones, Chip, Memory, Dram, Chips, Semiconductor, NAND, Foundry, Huawei	Semiconductor Industry News
8	166	Tribune, Editorial, Americans, Governors, Columnist, Florida, Herd, Sick, Dangerous, Republican	US related news
9	150	Daenam, Cheongdo, Hospital, Woman, Patient, Pneumonia, Daegu, Spreader, Patients, Old	Daenam Hospital Cluster Coronavirus Cases
10	148	Clinical, Trials, Drug, antibody, Celltrion, Drugs, CT, Phase, Treatments, Pipeline	Celltrion's Vaccine Development

**FIGURE 2.**
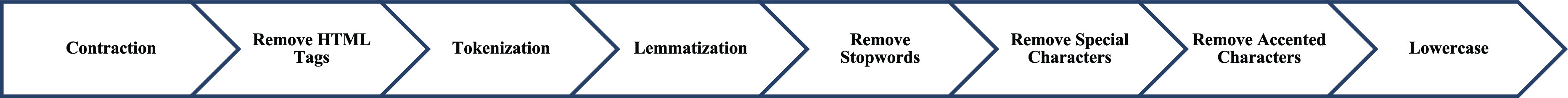
Preprocessing Steps Used in Cleaning Headlines.

**FIGURE 3.**
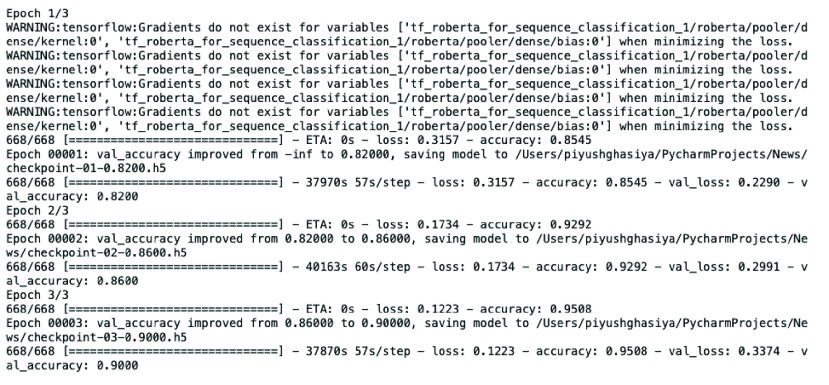
RoBERTa Sentiment Classification Model Summary.

**FIGURE 4.**
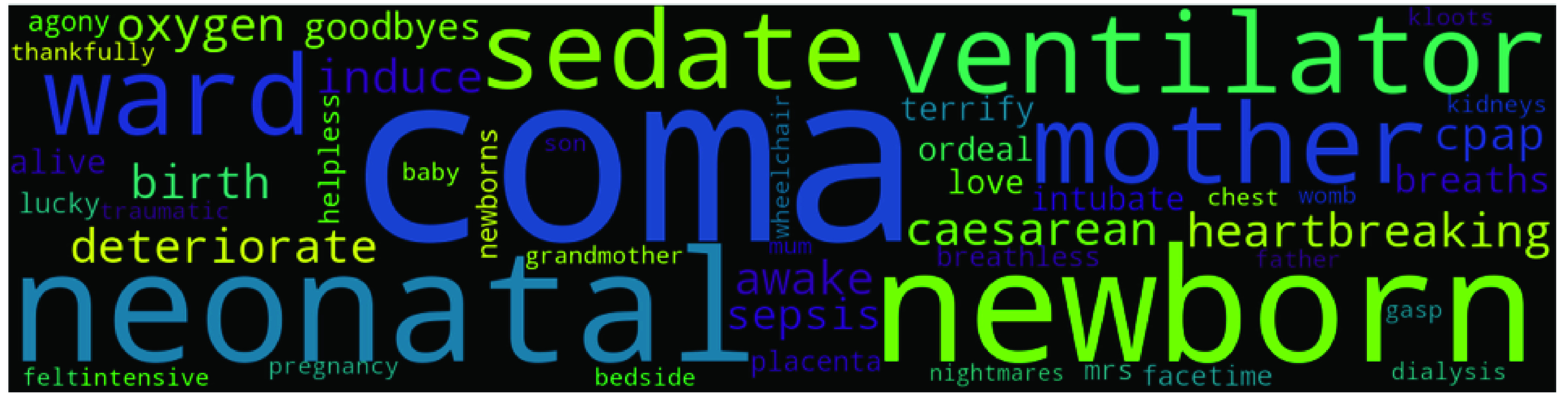
Topic 1 in UK’s Dataset - Maternity related news during pandemic.

**FIGURE 5.**
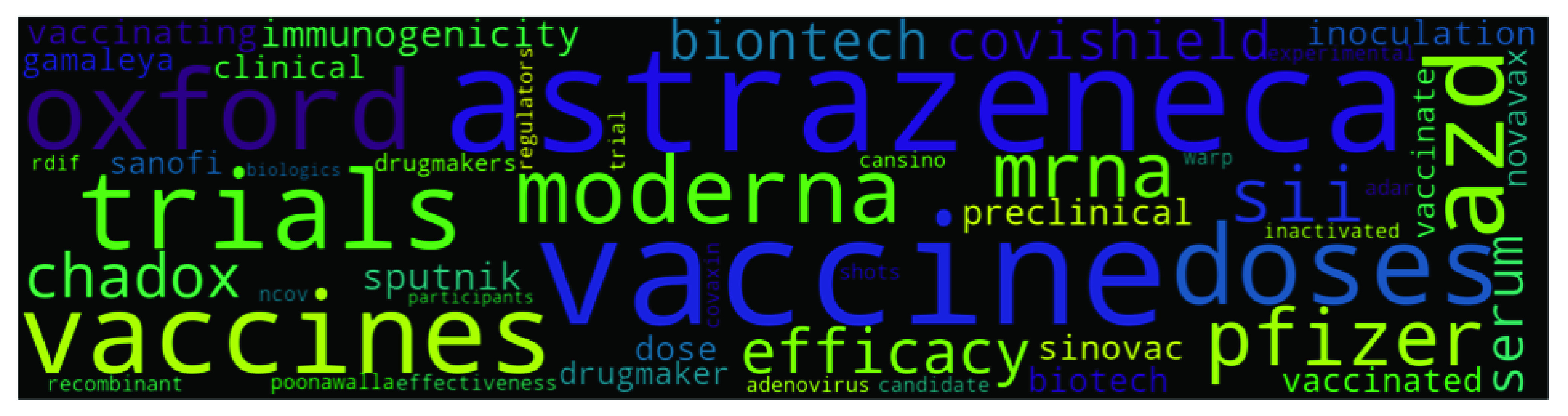
Topic 5 in India’s Dataset - Vaccine Development related News.

**FIGURE 6.**
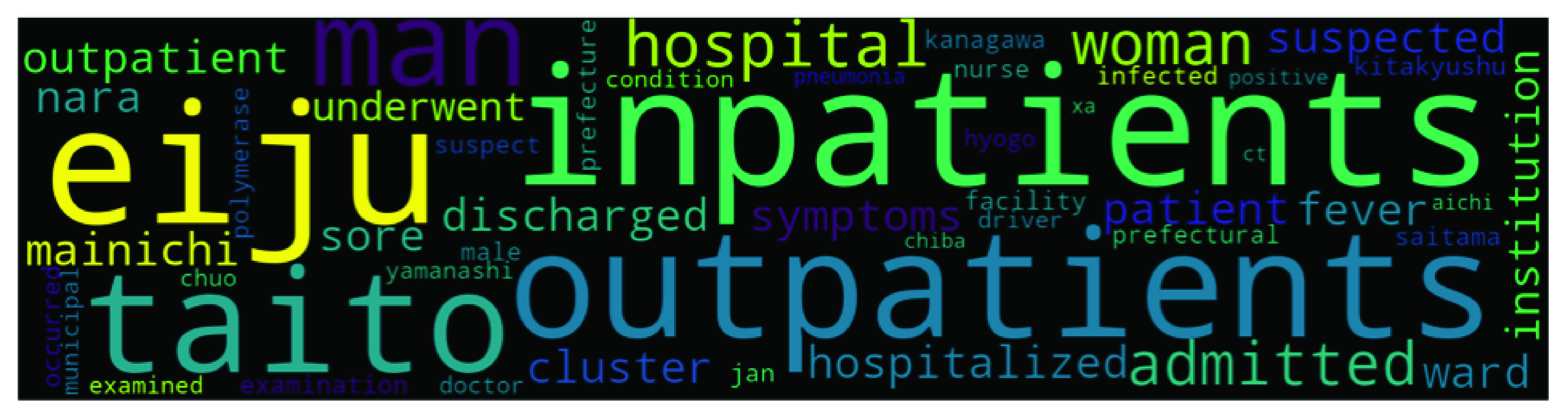
Topic 4 in Japan’s Dataset - COVID-19 Cases in Different Prefectures.

**FIGURE 7.**
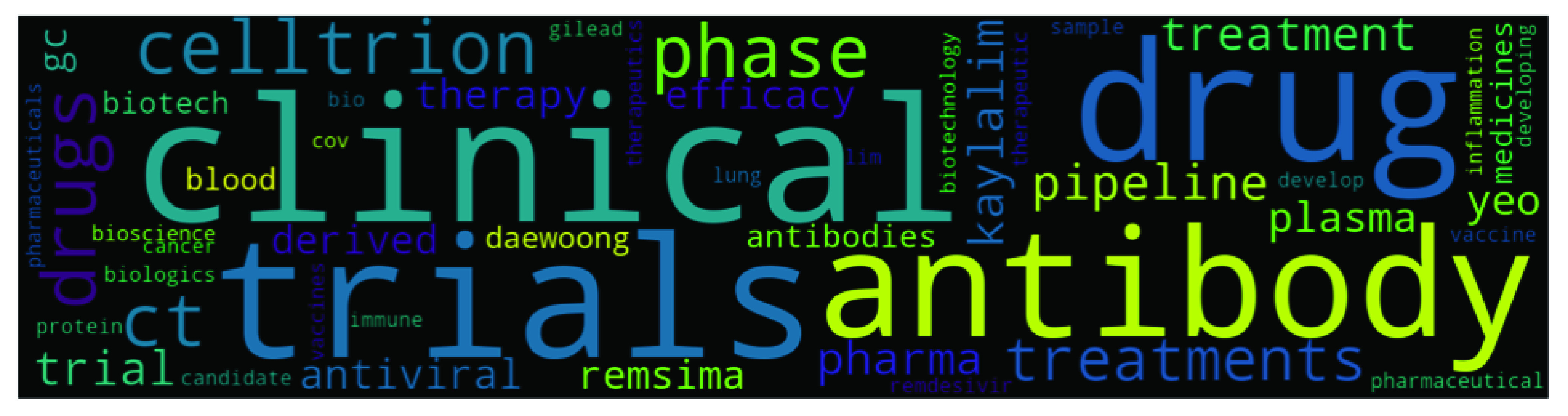
Topic 10 in South Korea’s Dataset - Celltrion’s Vaccine Development related topic.

### C. Preprocessing

In NLP, text preprocessing is the practice of cleaning and preparing text data. Following are the steps which we applied to preprocess the headlines.

1) Contractions are shortened version of words like don’t or I’d. In the first step, we expanded all these words into their original form.
2) Removed HTML tags.
3) Tokenization of headlines.
4) The root word is also known as lemma. With this step, we converted all the token into their root word.
5) Removed all the stopwords using NLTK library.
6) Special characters are usually non-alphanumeric characters, which can add noise to the text. So, we removed the special characters.
7) Since we are analyzing English language headlines, we need to make sure that all the accented characters are converted and standardized into ASCII characters.
8) Lastly, we converted all the words into lower case.

Fig. 2 shows the preprocessing steps we used to clean the news headlines for sentiment classification.

### D. Labeling Headlines for Sentiment Classification

Majority of sentiment analysis research is being conducted on Twitter or other social media posts. Because these posts tend to be highly subjective which makes them a good resource for sentiment analysis. Whereas news and news articles present facts which make them more objective (expect editorials and opinion pieces). In any supervised machine learning approach, labeled data is extremely critical and it is very difficult to manually verify the labels on large news articles. Owing to this reason we chose headlines. The first step to label the headlines for this research is: using unsupervised sentiment analysis method. We used three most popular python-based libraries 1) VADER [35], 2) Textblob [36], and 3) SentiWordNet [37] on 102,124 headlines.^2^ The second step is: to keep headlines that are categorized by all three library as positive and negative. We discarded all the headlines which are categorized differently by these three libraries. For example: if a headline is categorized as positive by VADER but negative by Textblob and SentiWordNet, then we discarded it from the labeling process. For this process we wrote a simple python code. After the second step we were left with only around 15% of the total headlines. In the third step, we manually confirmed all the headlines from the second step. Lastly, we used oversampling to balance (almost) the labeled data. Oversampling can lead to overfitting. One of the ways to find of whether model is overfitting or not is to check difference between training accuracy and validation accuracy. The training accuracy of the 3rd (last) epoch of our classification model (which gave 90% validation accuracy) have 95.08%. As the difference between training and validation accuracy is not very high, it can be said that our model is not overfitting. Fig. 3 shows our sentiment classification model summary. In this way, we collected 10,727 headlines (5369 positives and 5358 negatives). This labeled dataset is used to fine-tune the RoBERTa model. Table 2 shows few samples of labeled headlines.^2^During the collection of the dataset, some headlines were missing or were in other languages (Korean). Because of this reason, the number of collected headlines are less than (154) the collected articles.

### E. Sentiment Classification With RoBERTa

The traditional bag-of-words based NLP classifiers such as LogisticRegressionCV, LinearSVC, and Naïve Bayes discard word order (hence context) and in turn the meaning of words in the document (semantics). However, context and meaning are very crucial to the problems of NLP. On the other hand, Bidirectional Encoder Representations from Transformers (BERT) [38], Robustly Optimized BERT Pretraining Approach (RoBERTa) [39] XLNet, GPT-2, and GPT-3 are some of the example of the pretrained model based on Transformers – a deep learning model introduced in 2017 [40]. RoBERTa is an extension of the original BERT Model. All these transformers based models takes into account the context for each occurrence of a given word. This is one of the reason behind the better performance of these models compared to the classical bag-of-words based models. The original BERT model produced state-of-the-art results in many NLP tasks. However, there was still room for improvements in training objectives, duration of the training, and the data on which it is trained. Hence the Facebook AI Research (FAIR) proposed an ”optimized” and ”robust” version of BERT. Compared to BERT, RoBERTa is trained on 1) much larger datasets, 2) much longer, 3) longer sequences, 4) with dynamic mask generation, 5) without Next Sentence Prediction (NSP) objective, and 6) larger batches.

## IV. Experiment, Results and Analysis

### A. Topic Modeling Results and Descriptive Analysis

As mentioned before, the top2vec model does not require preprocessing and provides the option to either train a top2vec model on your dataset or use a pre-trained model universal-sentence-encoder. For this research, we trained the top2vec model on our collected dataset of each country individually. Further, top2vec has three parameters to determine the speed - fast-learn, learn, and deep-learn. Fast-learn is the fastest but provides the lowest quality results, while deep-learn takes the longest time to train but provides the best results. We trained our top2vec model on deep-learn parameters. Table 3 shows the results of the top2vec model on our dataset.

Indian dataset with the largest number of articles produced the highest 402 topics, while the South Korean dataset with the lowest number of articles came up with 127 topics. Following is the description of each of the four countries’ top topics and comparative analysis.

#### 1) Describing UK’s Top Topics

UK’s dataset produced 308 topics, and the biggest topic (369 articles) was about maternity during COVID-19 pandemic related news in the UK. The other top topics are Education, Australia, New Zealand, US, Office of National Statistics (ONS), economy, unemployment, and panic buying. The presence of Australia (topic 3) and New Zealand (topic 8) (commonwealth nations) and two topics related to the US (topic 4 and 6) (worst affected country) related news in the top 10 shows the special relationship between these countries and the UK. There was a huge impact of the COVID-19 pandemic on education worldwide, and UK is no exception to that. This fact is confirmed by the presence of education-related news in the second position. As a foremost UK’s statistics authority, ONS is leading an effort in managing COVID-19 data. Hence, it is one of the most talked-about government agencies in UK during the COVID-19 pandemic. Table 4 present the top 10 topics with the number of articles (topic size), top 10 words, and topic label for the UK. Fig. 4 shows the word cloud of the biggest topic – Maternity during the COVID-19 pandemic in UK’s dataset.

#### 2) Describing India’s Top Topics

When we trained the top2vec model on an Indian dataset of 47,342 articles and got 402 topics, the biggest topic with 1278 articles was Cricket and Indian Premier League (IPL). IPL is a 20/20 cricket league which has gained immense popularity and commercial success in India. However, it was postponed due to the pandemic and was later organized in UAE. Other top topics in the Indian dataset were - US, Punjab, Crimes and law & order cases, Education (related to schools on topic six, and University-related on topic nine), Vaccine development, Reserve Bank of India (RBI) economic policies, and COVID-19 testing. US presidential election was planned for November 2020, and along with the COVID-19 pandemic, the election campaign was also going on. The US is the leading global power, and its election results impact the whole world. Because of this reason, global media keeps a close eye on the US election, and Indian media is no exception to that. Indian drug companies are major manufacturers of vaccines distributed worldwide and are supplying more than 60% of the vaccines to the developing world [41]. Hence, it is understandable to see vaccine development related news in the top ten topics. Table 5 presents the top 10 topics with the number of articles (topic size), top 10 words, and topic label for India. Fig. 5 shows the word cloud for the 5th biggest topic – Vaccine development.

#### 3) Describing Japan’s Top Topics

Our top2vec model came up with 255 topics in Japan’s dataset. News about global stock exchanges and markets such as Dow Jones and NASDAQ were widely reported in Japanese media during this pandemic especially and came upon the top spot with 409 articles. Other top topics include Nikkei (Japan’s stock exchange) News, postponement of Tokyo 2020 games, North Korea (topic nine), South Korea (topic five), US election, and education-related news. The presence of stock markets news (global market in the first position and Nikkei on the second position) shows Japan’s English language media is paying too much attention to markets and trade even during the pandemic. Japan has invested a huge sum of money in preparing for Tokyo 2020 games and expected considerable economic benefits from it, but due to pandemic Tokyo 2020 games were postponed, this was a huge setback for Japan. Tokyo 2020 games postponement issue came up in the third position with 363 articles. South Korea is an important neighbouring country for Japan. Hence, during the pandemic, South Korea’s news was widely reported in the Japanese media and came on the fifth position. In contrast, North Korea is seen as a threat by Japan and it keeps a close eye on its activity. Top 10 topics with topic size, top words, and topic labels are shown in Table 6. At the same time, Fig. 6 shows the 4th biggest topic in Japan’s dataset.

#### 4) Describing South Korea’s Top Topics

Our COVID-19 dataset has the lowest number of articles (10,076) from South Korea, and this produced the lowest number of topics - 127. News related to budget and economic relief package provided during the pandemic in South Korea came up at top position with 284 articles. Other top topics include – the pandemic’s impact on retail and markets, geopolitics, low GDP forecast, sports, US, and vaccine trials. Out of the top ten topics, four topics (topic one, three, four, and seven) are economy-related in the South Korean dataset. This result shows South Korean media’s excessive attention to economy-related issues during the pandemic. Table 7 shows the top ten topics in the South Korean dataset and Fig. 7 shows Celltrion’s Vaccine development related topic (topic ten).

#### 5) Comparative Analysis

From our observation of Table 4, 5, 6, and 7, we found out that there are some common topics that are present in the top topics of each country. These topics are - US, Economy, Education, and Sports. For example, the US-related topics present in - UK (topic four and six), India (topic two), Japan (topic six), and South Korea (topic eight). Similarly, economy-related topics present in – the UK (topic seven and nine), India (topic seven), Japan (topic one and two), and South Korea (topic one, three, four, and seven). Table 8 shows these common topics. We can infer that economy, education, and sports are the most affected sectors by the COVID-19 pandemic. At the same time, the US’s presence at the top position in every dataset has two-fold implications. One - the worst affected country by COVID-19 pandemic and second – the US presidential election’s significance for the world. We would use the comparative analysis results in our second step of this research (sentiment classification) to investigate how the news sentiments of these common issues vary in all four countries.

**TABLE 8:** Common Topics in Top Position

	US	Economy	Education	Sports
Britain	✔	✔	✔	✘
India	✔	✔	✔	✔
Japan	✔	✔	✔	✔
South Korea	✔	✔	✘	✔

### B. Sentiment Analysis Results and Analysis

To perform sentiment classification, we used our labeled dataset of 10,727 headlines. We fine-tuned the RoBERTa BASE model with 12 layers and 768 hidden dimensions. We setup hyperparameters of 3 epochs, the learning rate of 1e-5, and a batch size of 16. The last third epoch achieved the validation accuracy of 90%. We also performed comparative tests using traditional bag-of-words approach based classifiers such as LinearSVC, MultinomialNB, BernoulliNB, LogisticRegressionCV, and others. Table 9 shows the accuracy of RoBERTa and different classifiers.

**TABLE 9:** Comparison of RoBERTa and Other Classifiers

System	Accuracy(%)
RoBERTa BASE	90
LinearSVC	92
MultinomialNB	88
BernoulliNB	88
LogisticRegressionCV	92
PassiveAggressiveClassifier	94
NearestCentroid	72
AdaBoostClassifier	70
Perceptron	94

We can see that there are few classifiers such as LinearSVC (92%), LogisticRegressionCV (92%), PassiveAggressiveClassifier (94%), and Perceptron (94%) that achieve high accuracy than the RoBERTa model. However, even with little less accuracy, RoBERTa was able to classify better than these classifiers. Table 10 shows few headlines (from the Indian dataset) where RoBERTa classified correctly, and other classifiers failed.

**TABLE 10:** Examples of RoBERTa and Other Classifiers Predictions

Headlines	RoBERTa Predicted Sentiments	Other Classifiers Predicted Sentiments
Asian shares slip on faltering hopes for COVID vaccines	Negative	Positive
COVID-19 infection offers protection from reinfection for at least 6 months: Study	Positive	Negative
Maharashtra: Counterfeit masks using leading brand's name major concern for manufacturer; cases, raids in many cities	Negative	Positive
Travel restrictions challenge vaccine rollout, airlines warn	Negative	Positive
Crude oil prices extend gains on COVID vaccine hopes, OPEC+	Positive	Negative

It is clear from the above Table 10 that RoBERTa performed better. It might be possible that for some headlines the traditional classifier may classify correctly compare to RoBERTa. The study of this possibility and it’s possible explanation can be a separate research topic in itself. Studies that specially focus on the comparative analysis of traditional and the transformer based sentiment classification can investigate it further. Presently, transformer based models have produced superior results in various NLP tasks and considered as the state-of-art. Because of this reason, we opted for RoBERTa to predict the sentiment of our whole dataset. Following is the detailed description of sentiment classification in all four countries individually.

#### 1) Sentiments of UK’s Headlines

**FIGURE 8.**
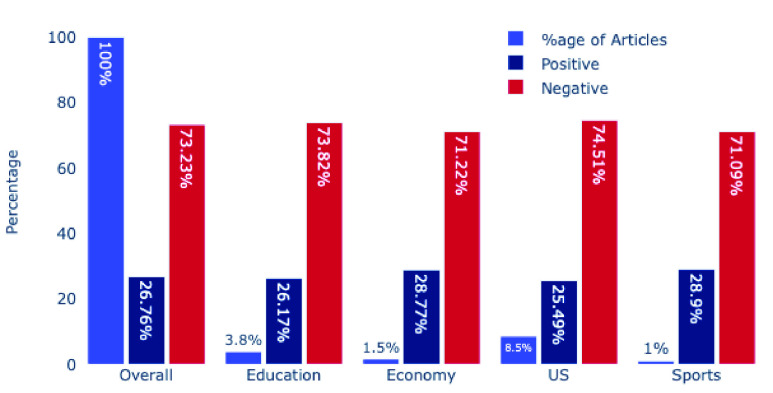
Overall and Various Topic’s Sentiment in UK’s Dataset.

Overall, out of 23,821 headlines, 17,445 (73.23%) were negative, and the remaining 6376 (26.76%) were positive. This result shows high negative sentiments in UK media about the COVID-19 pandemic and other related issues. When we investigated the sentiments of the topics that were common in all four countries based on (Table 8), we found out that the US has more negative (74.5%) than the overall percentage. In comparison, sports were the least negative (71.09%). Fig. 8 shows overall and various topic’s sentiment of UK’s dataset. It is clear from Fig. 8 that the various other topics’ sentiments almost (slight fluctuation) follow the trend of the overall sentiments in the UK’s dataset.

#### 2) Sentiments of India’s Headlines

**FIGURE 9.**
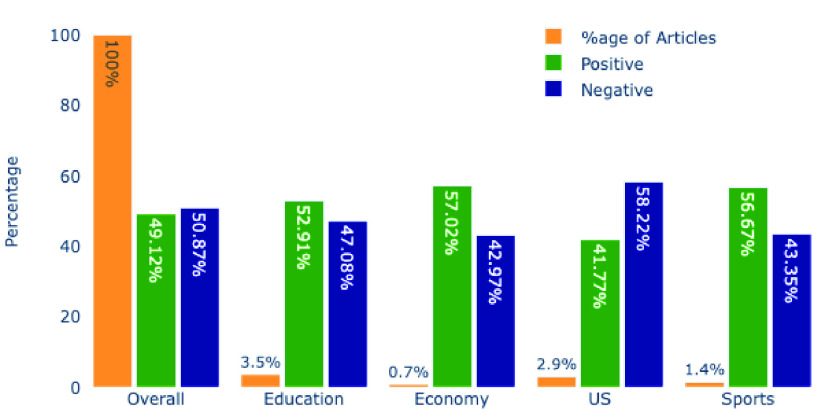
Overall and Various Topic’s Sentiment in Indian Dataset.

Indian dataset came out to be almost balanced. Overall, the Indian dataset has 24,017 (50.87%) negative and 23,193 (49.12%) positive headlines. When it comes to the sentiments of various topics, all of the topics followed a different trend than overall sentiments. US related headlines have the highest percentage of negative with 58.22%. In contrast, the Economy category has 57.02% positive headlines. In Fig. 9 we can see various topics’ sentiments in the Indian dataset.

#### 3) Sentiments of Japan’s Headlines

**FIGURE 10.**
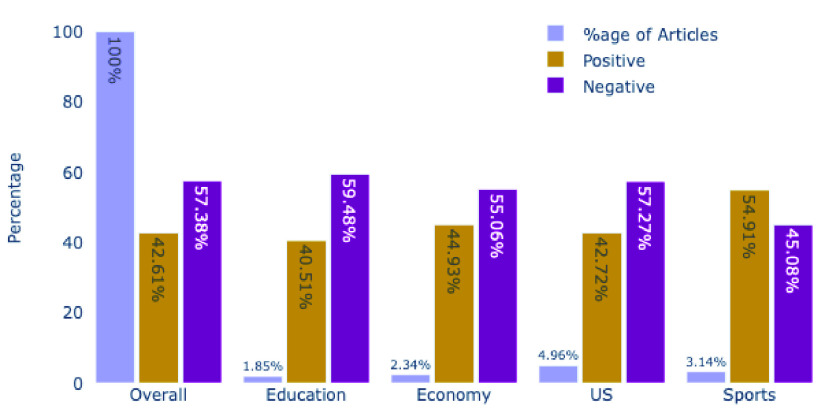
Overall and Various Topic’s Sentiment in Japan’s Dataset.

Japan’s dataset turns out to be more negative tilted. As out of 21,038 Japan’s headlines, 12,073 (57.38%) were negative, and the remaining 8965 (42.61%) were positive. Sports turns out to be following a different pattern than rest of the topics. All topics excluding sports, were in range 42% ± 2% (for positive) and 57% ± 2% (for negative). While the sports category has 54.91% positive and 44.08% negative headlines. On the other hand topic of education received the most negative (59.48%) than any other topic. Fig. 10 shows sentiments of various topics in Japan’s dataset.

#### 4) Sentiments of South Korean Headlines

**FIGURE 11.**
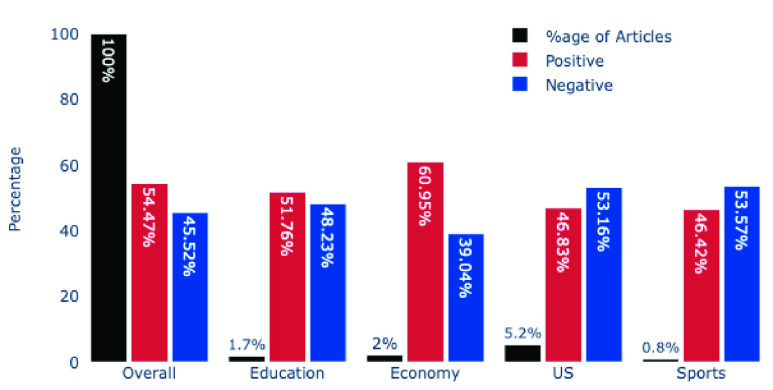
Overall and Various Topic’s Sentiment in South Korean Dataset.

Out of 10,055 headlines in our South Korean dataset, 54.47% (5477) were positive, and the remaining 4578 headlines (45.52%) were negative. These results make the South Korean dataset most positive among the four nations that we investigated. When we inspected various topics’ sentiments, we found out that the economy-related topic was the most positive, with 60.95% of the headlines being positive. In comparison, the US and sports topics turned out to be the most negative with 53.16% and 53.57% negative headlines, respectively. Fig. 11 presents the sentiments of various topics in the South Korean dataset.

#### 5) Comparison of all Four Nations Sentiments

**FIGURE 12.**
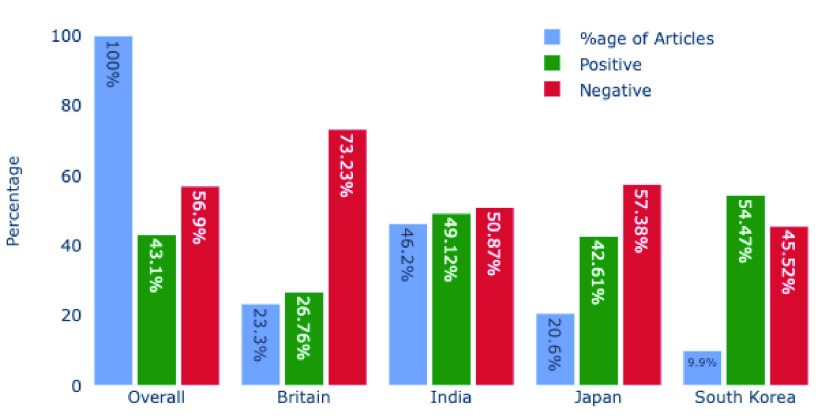
Comparison of Countries COVID-19 News Headlines Sentiments.

When we evaluate our complete COVID-19 dataset of 102,124 headlines, we discovered that 56.9% (58,113 headlines) were negative, and the rest of 44,011 headlines (43.1%) were positive. These results show (not surprisingly) that overall there was more negative news about the COVID-19 pandemic. UK and South Korea are the most negative and positive country, respectively. UK has almost three-quarters of news (73.23%) that were negative. In comparison, South Korea has 54.47% of positive news, which is more than 10% higher than the overall percentage. Interestingly, out of the four countries that we studied, UK is the worst affected country (in terms of deaths per million - 1320). At the same time, South Korea is the most successful country to tackle the COVID-19 pandemic, with only 25 deaths per million [42]. India is the only developing country in our research. While in terms of infected people, India is the 2nd worst affected country after the US, but when taking deaths/million into consideration, it is better than most developed countries. Our results show that the Indian dataset is the most balanced dataset with 50.87% negative and 49.13% positive headlines. Fig 12 shows the comparison of all four countries sentiments.

## V. Limitations

Though we analyzed more than 100,000 news articles related (directly or indirectly) to COVID-19 from four countries, however, it is impractical to cover all the facets of such a vast domain. This section presents the limitations of our work.

1) Though the number of topics generated by top2vec are numerous (1092 if we combine topics from all four countries). In this research we have taken into consideration only the top ten topics from each country. Further, topic labeling is a manual process. Hence, topic label may differ based on the author’s understanding and knowledge of various themes and issues.
2) Supervised machine learning requires labeled dataset to train and test the model. We have created our own labeled dataset of COVID-19 news headlines partly using unsupervised methods and partly by manual verification. There is enough possibility to further improve this labeled dataset.
3) Sentiment classification has two variants. Binary, where sentiment is classified as positive and negative, while the other one is multi-class where text sentiment can be divided into more than two classes. For example: three classes where sentiment is classified into positive, negative, and neutral. This research has focused in classifying news headlines into either positive or negative classes (binary). However, certainly there is a possibility that multiclass sentiment classification (positive, negative and neutral) may produce better results.
4) In this research we have collected COVID-19 related news articles either from digital English language newspaper (for UK and India) or from the newspaper’s English website (for Japan and South Korea).
5) Collection of data also faced limitations such as paywall restrictions, change of website format, and other web-scraping related issues. Also, we have only used two keywords: Coronavirus and COVID-19 to collect news articles.

## VI. Conclusion and Future Work

Even after almost a year since it started, the COVID-19 pandemic is still unstoppable. Despite having many promising vaccine candidates, including some who have been given approval for emergency use in various countries, it is still difficult to say when the vaccine would be available for common people. Keeping this situation in mind, in our research, we tried to discover and comprehend the critical issues and sentiments of COVID-19 related news. Our topic modeling experiment and analysis shows that the US, Economy, Education, and Sports are among the most widely reported issues in all four countries, while UK has the most negative COVID-19 related news.

Our study collected more than 100,000 COVID-19/Coronavirus related news articles from four countries for 11 months. In the first part of this research - topic modeling - we used the top2vec model and produced topics for each country. The number of topics turns out to be directly proportionate to the number of articles, as India, with the highest number of articles (47,342), produced the highest number of topics (402). Further, our descriptive analysis of the top ten topics across all four nations showed that the US, Economy, Education, and Sports are the most common issues. The presence of the US in the top ten in all countries shows the significance of the US. While education, economy, and sports can be seen as the worst affected sectors during this pandemic.

The next part of this research utilized the state-of-the-art RoBERTa model for sentiment classification of headlines. Our model achieved 90% validation accuracy and was able to classify headlines better than other traditional classifiers. Our implementation of the RoBERTa model for sentiment classification on our complete dataset showed that UK has the most negative news (73.23%), while South Korea was the most positive country with 54.47% positive news. These results correspond with the real situation of the COVID-19 pandemic in these countries as UK is one of the worst affected countries in terms of deaths/million, and South Korea is one of the best-performing countries with only 15 deaths/million. With these results, this study can be used as a template to study the COVID-19 news worldwide, helping us discover and understand the critical issues and their representative sentiments in COVID-19 news in those countries.

Other than the above mentioned results, this analytical study helps us understand the nature of newsmaking. By looking at the topics in different countries, we can see that even COVID-19 news reporting were based on the overall national interest and strategic significance. For example: presence of the US in all of the four countries, Australia related news in the UK media, North and South Korea related news in Japan’s media. Our research also highlights the fact that some of the worst affected sectors (education, economy, sports) are also the most widely reported. Our sentiment analysis result also points out towards a possible correlation between negative COVID-19 news and a countries’ affectedness. For example: In our dataset, UK is the worst affected country and also has the highest negative headlines. This correlation can be further tested by deploying the same or improved methodology by other researchers. The results and subsequent analysis can also be beneficial for researchers who are trying to understand the newsmaking process or the cross-cultural socio-political-economic COVID-19 impact. While majority of the COVID-19 sentiment analysis researches are focused on social media posts, our study presents an alternative perspective to this by introducing COVID-19 news headlines sentiment analysis.

In future works, first we would try to overcome the limitations of this study. This includes further research on comparative analysis of all topics from all four counties and multi-class sentiment classification. We would also try to improve the accuracy of our RoBERTa model. New and interesting methods have been proposed to detect emotion [21] and sentiment [22] by using models such LTSM and lexicon-based convolutional neural network. We would also try to explore these models in our future studies. Lastly, we would try to add more countries in our dataset to enlarge the scope of this research in order to make it more wide-ranging and global.

## Supplementary Material

10.21227/gdq8-ej60 COVID-19 News Articles
